# Ecophysiology of the conjugating green algae (Zygnematophyceae) with emphasis on semiterrestrial habitats and specialized metabolic compounds

**DOI:** 10.1007/s00709-026-02204-y

**Published:** 2026-05-01

**Authors:** Daniel Remias, Lenka Procházková

**Affiliations:** 1 Department of Environment & Biodiversity, University of Salzburg, Salzburg, Austria; 2 Faculty of Science, Department of Ecology, Charles University, Prague, Czech Republic

**Keywords:** Phycology, Terrestrialisation, Streptophytic algae, Phenolic pigments, Aerophytic

## Abstract

The conjugating green algae (Zygnematophyceae) are regarded as sister group to land plants. Despite their morphological simplicity, recent genomic and other “omics” breakthroughs revealed a molecular toolkit for terrestrial survival that was established before the rise of embryophytes. This mini-review synthesizes advances by focusing on extreme habitats like glaciers, acidic peat bogs and aeroterrestrial surfaces. We highlight how environmental pressures have driven adaptations, including: (a) Genomic and cellular plasticity: substantial genome size variation and horizontal gene transfer events from soil bacteria that enhance metabolic flexibility. (b) Resistant life stages: the formation of zygospores and pre-akinetes with complex, multi-layered cell walls. (c) Specialized metabolites: the accumulation of unusual phenolics in vacuoles, cell walls or their excretion into the surroundings. These compounds, sometimes complexed with iron, function as photoprotectants against excessive UV and visible radiation but may have further roles as well. We address the lack of cultures to study zygospore formation and germination for life cycle studies, and the general under-sampling of “orthodox” habitats, where particularly members of the Serritaeniales and Zygnematales prevail. Resolving the phylogeny and chemical characterization of these algae is essential to understanding the ancestral stress-response mechanisms that enabled the colonisation of land.

## Introduction

The Zygnematophyceae (also known as Conjugating Green Algae, CGA) are a distinct and diverse lineage of freshwater microalgae (Gontcharow 2008, Büdel et al. 2024). They belong to the Streptophyta, and they are recognized as a sister group to land plants (Wickett et al. 2014; De Vries and Archibald 2018; Rensing 2020). Interestingly, CGA exhibit high morphological simplicity (unicellular or unbranched filaments, a lack of any tissues or complex diploid stages), unlike other streptophytic algae like macroscopic stoneworts (Charales). This phenomenon is thought to be the result of a secondary reduction that developed over a long period of cold, unfavourable climatic conditions on Earth (Žárský et al. 2022).

In single-cell genera, morphology and environment are closely linked, e.g. in *Micrasterias*, the branching pattern of the cells clearly correlated with the phylogeny, whereas cell complexity possibly reflects their adaptive morphological responses to environmental conditions (Škaloud et al. 2011). A recent genomic analysis of signalling networks by Feng et al. (2024) further illuminates the above mentioned “evolutionary paradox.” Their work details how environmental response genes (e.g., to desiccation and light stress) were expanded in the last common ancestor of Zygnematophyceae and embryophytes. These findings confirm that the molecular “toolkit” for terrestrial survival—including complex cell wall biosynthesis—was largely assembled in these algae long before Embryophyta began their conquest of land. This is further reflected in the chemical capacity of charophyte algae (including Zygnematophyceae) to produce key phytohormones, such as auxin and salicylic acid, ethylene, and t-RNA derived cytokinins although they lack the complex hormone conjugation systems and *trans*-zeatin type cytokinins found in land plants (Schmidt et al. 2024). Furthermore, the presence of a genetic repertoire for multicellular growth in contemporary unicellular CGA strongly supports the theory of a history of reductive evolution in this lineage, positioning these algae as key models for research of several aspects of evolutionary and cell biology (Ohtaka and Sekimoto 2023), including sex determination (Sekimoto et al. 2023).

**Fig. 1:**
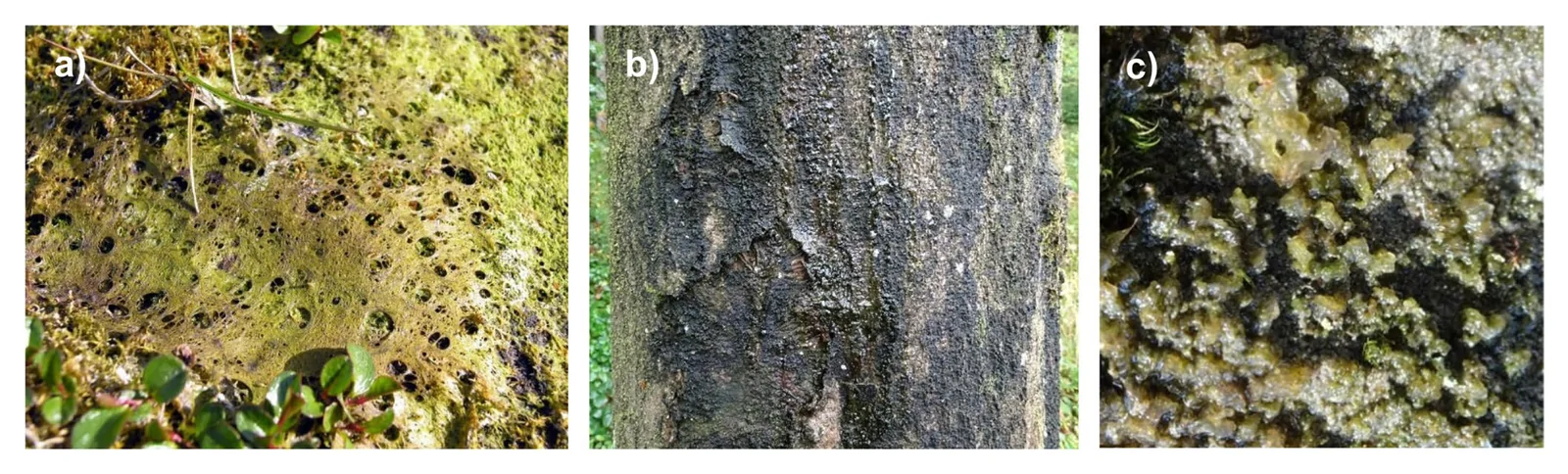
Examples of aeroterrestrial, macroscopic zygnematophycean blooms. (**a**) Crust-like mat on soil, mainly made of *Zygnema* sp, Svalbard. (**b**) dark mucilage caused by *Serritaenia* sp. on *Abies* tree bark, Austria. (**c**) greenish mucilage caused by *Mesotaenium chlamydosporum* var. *violascens* on wet rocks, Eastern France

Generally, algae are very common in all aquatic eco-systems including the seas, ponds and rivers. CGA likely originated in freshwater habitats, and they can particularly dominate microbial communities in exposed or extremophilic environments (Fig. 1), which will be the focus of this mini review. For instance, they frequently occur in very acidic, shallow peat bogs, on moist aeroterrestrial surfaces like rocks, open soils, on mosses, leaves and bark-surfaces (Busch and Hess 2022a), and even on bare melting glacier ice (Hoham and Remias 2020). These blooms at exceptional sites indicate certain molecular and physiological adaptations to abiotic stress, a development which could be considered as a prerequisite for colonising semi-terrestrial habitats and eventually the land.

On the whole, the terrestrialisation of photoautotrophs on Earth required numerous biochemical adaptations, because life above the water surface includes abiotic challenges like water scarcity, low nutrients, an oxygenic atmosphere, excessive visible (VIS) or harmful ultraviolet (UV) solar irradiation and significant short-time temperature and irradiance fluctuations (Fürst-Jansen et al. 2020; Permann et al. 2022a). To cope with these challenges, algae have evolved a variety of stress response mechanisms, including the production of specialized metabolites, desaturation of membrane lipids, activation of reactive species scavengers or the accumulation of compatible solutes (Kaur et al. 2022).

## Biodiversity and Distribution

The CGA comprise more than 8800 species living in all kinds of freshwater habitats, and were traditionally divided into two orders, the Desmidiales and Zygnematales (Lewis and McCourt 2004; Zhou and von Schwartzenberg 2020). However, a comprehensive phylogenomic analyses of Hess et al. (2022) showed the polyphyly of both, and currently three additional orders, the Spirogyrales, Spirogloeales and the Serritaeniales, are accepted. In addition, Cheng et al. (2019) used molecular methods to identify one of these new lineages in the Zygnematophyceae, naming it as subclass Spirogloeophycidae. Despite the many indications of polyphyly in various traditional taxa of the CGA (Gontcharov 2008), their monophyletic origin (with the exception of *Spirotaenia* falling into three clades, Renner and Sokoloff 2024) and phylogenetic position as a sister group to embryophyte land plants is unquestioned (Wickett et al. 2014).

As mentioned above, various CGA species have been reported or described in unusual habitats in both cold and warm regions of the Earth. This highlights the fact that many habitats in remote areas have not yet been adequately sampled. For example, spectacular reddish blooms dominated by algae morphologically identified as *Mesotaenium macrococcum* and *Mesotaenium chlamydosporum* were reported by Montoya et al. (2022) from rocks in montane cloud forest in the Alccamayo ravine in the tropical Peruvian Andes (from 2184 to 3000 m a.s.l.). A remarkable extremophilic species was described by Garduño-Solórzano et al. (2021) from a high-altitude (4500 m), oligotrophic and UV-exposed mountain lake in Mexico. There, the filamentous *Temnogametum iztacalense* sp. nov. (Zygnemataceae) formed macroscopic, purple mats in the bottom of the lagoon, and the striking secondary pigmentation was located in abundant, purple-coloured vacuoles. Well-known genera such as the *Micrasterias* have shown unrevealed diversity, which recently resulted in the erection of the new desmid genus *Pseudomicrasterias* gen. nov. by de Araújo et al. (2022), based on samples from tropical Brazil. CGA are widespread not only in warm, but also cold regions: Melting glacier surfaces in mountainous and polar regions harbour dark surface blooms mainly caused by psychrophiles of the genus *Ancylonema* (Remias et al. 2012a). In this case of living permanently on ice, cells possess abundant darkly pigmented vacuoles, similar to *Temnogametum*. Furthermore, molecular analysis has shown that the unicellular glacier ice alga formerly known as “*Mesotaenium berggrenii”* belongs to the genus *Ancylonema* (Procházková et al. 2021). Metagenomics of Antarctic ice fields has revealed further, yet undescribed cryophilic *Ancylonema* species (Thomson et al. 2025). Interestingly, Busch et al. (2024a) described with *A. palustre* the first mesophilic species of *Ancylonema* from swamps in Germany, and the cells had dark pigmented vacuoles like glacier ice species of the same genus. At the Arctic archipelago of Svalbard, Barcytė et al. (2020) discovered seven morphologically *Cylindrocystis*-like species living in soil and on glaciers, but by molecular means they turned out to belong to two different clades, thus indicating the polyphyly of this genus.

These examples highlight a yet undiscovered biodiversity and some unresolved relationships within the CGA. Morphological similarities within many traditional ‘saccoderm desmid’ genera, such as *Mesotaenium* (Busch and Hess 2022b), and ‘placoderm desmid’ genera, such as *Cosmarium*, can explain this current situation. These genera are no longer considered to be monophyletic (Hess et al., 2022). However, there is also a clear lack of consistent sampling of cells, apart from well-known benthic sites, in otherwise “orthodox” habitats such as rock surfaces, mosses, tree bark or damp soil (Busch and Hess 2022b).

## Cultivation and sequencing

Many described CGA taxa have never been cultured, and existing strains in accessible collections cover only roughly 8% of all known species (Zhou and von Schwartzenberg 2020). This may be partly because many algae from extremophilic habitats resist cultivation. The authors recently established, for the first time known in the scientific community, a strain of a glacier ice alga from Austria, *Ancylonema alaskanum* (Remias and Procházková 2023) while Jensen et al. (2023) established in a similar manner a strain from the Greenland Ice Sheet, *Ancylonema* sp. Furthermore, by selecting the hypervariable ITS2 marker, the Remias and Procházková (2023) introduced a method with sufficient sensitivity to distinguish any kind of CGA at the species level. Consequently, molecular biodiversity studies including Zygnematophyceae can now be conducted at a higher resolution, e.g. to target non-culturable species in the field (Remias et al. 2023).

Meta-barcoding (high throughput sequencing) has revealed the presence of CGAs in various habitats and geographic regions. For example, case studies on biological soil crusts, which are extremophilic habitats regarding desiccation and temperature conditions, reported the presence of CGAs (Holzinger et al. 2018; Rippin et al. 2019). However, various aeroterrestrial surfaces in forests (e.g., tree bark, evergreen leaves), rocks, moorlands and heathers remain under-sampled. Applying environmental sequencing to e.g. temperate and sub/tropical biofilms will be useful for increasing our knowledge of CGA biodiversity and to demonstrate their presence (and thus likely adaptation) to certain ecological niches.

## Morphologies, cytology and life cycles

The photosynthesis of vegetative pre-akinetes has been shown to be more impaired after UVB-exposure compared to young vegetative cells in species of *Zygnema* isolated from polar soil crusts (Holzinger et al. 2018). On the other hand, mature pre-akinetes were reported to show high frost tolerance (Trumhová et al. 2019). One of the most important life-cycle stage of CGAs in terms of coping with challenging abiotic conditions are the meiotic zygospores: The zygospore formation of *Spirogyra mirabilis* from an alpine habitat was described in detail by Perman et al. (2021a), demonstrating that these robust stages are significant for survival during unfavourable environmental conditions like heat or drought. Likewise, Stancheva et al. (2016) found similar principles for *Zygogonium ericetorum* living in a shallow high-alpine brook. Cytological insights into why zygospores become resistant were given for four different species by Permann et al. (2021a, b, 2022c, 2023): a combination of complex, three-layered and dark pigmented cell walls, an increase in lipid droplets, and a reduction of plastidal starch content. Additionally, Pfeifer et al. (2022) demonstrated the evolutionary proximity of CGA to land plants by finding rhamnogalactan-proteins in the cell walls of *Spirogyra pratensis*, while the wall of a stonewort did not have such structural supporting elements. Herburger et al. (2019) proved that homogalacturonans in the cell walls of *Zygnema* sp. increased desiccation resistance, indicating that polysaccharide-rich cell walls are a prerequisite for terrestrial survival. Another derived achievement of CGA is the active intracellular movement of chloroplasts depending on the light conditions. In particular, the phenomenon of chloroplast rotation has been observed for the genera *Mougeotia* and *Mesotaenium* (Permann et al. 2024; and references therein) and in the case of Spirogyra, chloroplast ribbon shifts (Ohiwa 1977). In addition to structural changes, the nuclear dynamics during germination are also specialized; as documented for *Closterium*, after meiosis and the subsequent degeneration of non-functional nuclei, the surviving gones may reach transiently a 4 C DNA content prior to their initial cell division (Hamada 1987). This likely represents a pre-loading of genetic material to facilitate rapid initial cell divisions and provide multiple templates for DNA repair after zygospore germination.

The CGA show significant variations at the genomic level. Kawaguchi et al. (2023) recently reported an extensive 2-fold heritable genome size variation in a *Closterium* species-complex caused by so-called Copy Number Variations of individual regions. The observed strong variation in genomes can be related to different ploidy levels (Poulíčková et al. 2014). Interestingly, such genome size variation can have various ecophysiological consequences. For instance, in microalgae, higher DNA contents can be coupled with physiological traits (bigger cell sizes, slower metabolic and growth rates or even with higher secondary metabolite production; Čertnerová and Škaloud 2020) and species distribution (Čertnerová et al. 2025). Unicellular microalgae are an ideal model for studying the ecophysiological consequences of genome size variation (Čertnerová and Galbraith 2021). Cell size can impact many traits including species ecology, light harvesting efficiency, efficiency of nutrient acquisition, susceptibility to herbivores and dispersal abilities etc. Previous studies have suggested that genome size may even have adaptive potentials due to its link with ecological traits (e.g. Trávníček et al. 2019). Nonetheless, the observed strong variations in genome size can be related to different levels of ploidy in life cycle of CGA (Renner and Sokoloff 2024).

## Habitat preferences as part of terrestrial colonization

Stamenković & Hanelt (2017) reviewed the geographical distribution of desmids in relation to abiotic factors. They emphasized the advantage of CGA’s high-light tolerance in comparison to other microalgae due to optimised photosystems and proposed that they originated in the tropical zone. There, like in temperate regions, CGA’s frequently occur in aeroterrestrial habitats such as on tree bark, as shown by Šťastný (2008), who identified an impressive number of 110 different CGA living in aeroterrestrial habitats in Central Europe. More recently, Sophia et al. (2022) found 57 subaerial CGAs in the Atlantic Forest of south-eastern Brazil. In tropical regions, several *Spirotaenia* species were found to live in so-called phytotelms, rainwater-filled cavities of vascular plants (Ramos et al. 2017). Nevertheless, most desmids *s.str*. settle in peat bogs, and filamentous Zygnematales usually grow in springs, rivulets or ponds.

## Zygnematophyceae in the age of “Omics”

The previous paragraphs illustrated how CGAs adapt to harsh abiotic conditions. However, the molecular mechanisms behind these adaptations are not yet fully understood. In recent years, the advent of Omics-protocols, focusing on e.g. metabolites, transcripts and genomes, has provided and will continue to provide new insights. Holzinger and Pichrtová (2016) reviewed the challenges of such techniques in streptophytic algae, highlighting their value as model organisms for studying stress tolerance connected with the transition to land. Noteworthy, Cheng et al. (2019) reported that a series of significant horizontal gene transfers that took place in subaerial Zygnematophyceae during evolution, sourcing from soil bacteria, and that these genes regulate plant growth and stress responses. Accordingly, the genome and transcriptomes of *Spirogyra pratensis* depicted activation of quiescence and homeostatic programs (Goldbecker et al. 2025). Dadras et al. (2023a) reviewed the role of secondary metabolism in the process of plant terrestrialisation. They argued that many specialized metabolites have rather acted as a pool of standing variation that was later co-opted for new functions during the conversion from green algae to land plants. The chemodiversity of phenolic compounds in streptophytic algae, primarily synthesized via the phenylpropanoid pathway, has enabled them to overcome selective pressures resulting from environmental stressors (Kunz et al. 2024). Phytochromes are photosensors that control physiological responses to red and far-red light, as shown by Wagner and Klein (1981) in a classical experiment for chloroplast rotation in *Mougeotia*. Li et al. (2015) found out that in streptophytic algae, phytochromes are structurally diverse, including canonical and non-canonical forms, while in land plants, the structure is highly conserved.

Desiccation tolerance is considered a vital characteristic for microalgae to colonise terrestrial habitats. For example, it has been demonstrated that liquid-grown *Zygnema circumcarinatum* algae are more susceptible to desiccation than those grown on an agar surface (Rippin et al. 2017). Furthermore, gene expression analysis revealed that photosynthesis was significantly suppressed only in liquid-grown cells. Conversely, stress protection mechanisms such as reactive oxygen species scavenging, DNA repair, and the expression of chaperones and aquaporins increased. Fürst-Jansen et al. (2022) investigated *Mougeotia* ssp. strains at the transcriptomic level before and after transferring the filaments from a terrestrial (solid agar medium) to an aquatic habitat (liquid medium). They identified numerous transcripts that were up- or down-regulated, which are responsible for photophysiological readjustment and carbon metabolism. For instance, the expression of one gene specifically linked to the light-harvesting complex showed a 5.8-fold increase, while the downregulation of a homolog of a gene encoding purple acid phosphatase suggested a shift in nutrient uptake or cell wall remodelling. Rieseberg et al. (2023) compared the molecular and morphological desiccation responses of two CGAs: *Zygnema circumcarinatum* and *Mesotaenium endlicherianum*. They identified transcript signatures associated with calcium-mediated signalling, as well as adjustments to plastid biology, cell envelopes, and amino acids. These reactions were similar to those observed in land plants. Light is another significant ecophysiological parameter: Mundt et al. (2019) investigated the gene expression patterns of the desmid *Cosmarium crenatum* in the Arctic over the course of one week of permanent darkness. They found strong repression of transcripts associated with photosynthesis, photorespiration, and cell wall development, while genes involved in starch catabolism were enhanced. Recently, Busch et al. (2024b) applied comparative transcriptomics to *Serritaenia*, finding that UV-exposure induced the repair of DNA and the photosynthetic apparatus as well as the synthesis of aromatic specialized metabolites. A combined approach of meta-transcriptomics and metabolomics showed a vertical heterogeneity in Arctic soil crusts made of *Zygnema* sp. and confirmed the hypothesis that cells of the top-layer protect the bottom layer from abiotic stress like excessive irradiation (Rippin et al. 2019). Exposure to very low temperatures as well as the occurrence of freeze-thaw-events damages algal cell and organelle membranes. Accordingly, the glacier ice alga *Ancylonema nordenskioeldii* typically living around freezing point temperatures, showed strong evidence for the expression of an DUF3494-type ice-binding protein (Procházková et al. 2024). By genome and transcriptome analysis, its ice-binding activity was confirmed using a freezing assay, i.e., the protein was secreted and eventually modified the recrystallization of ice in the cell’s surroundings (Procházková et al. 2024). Most recently, the so far most comprehensive metabolomic and transcriptomic study on a CGA included 42 different growth conditions and 128 transcriptomes applied to one strain (Dadras et al. 2023b): they stressed that landmark proteins of land plants were also present in the CGA, which underscores that aspects of plastid–nucleus communication are probably shared across more than 600 million years of streptophyte evolution. Moreover, they found a homologous program for a prominent, stress-dependent lipid droplet synthesis that existed prior to plants’ conquest of land.

## The striking phenolics of conjugating green algae

**Fig. 2:**
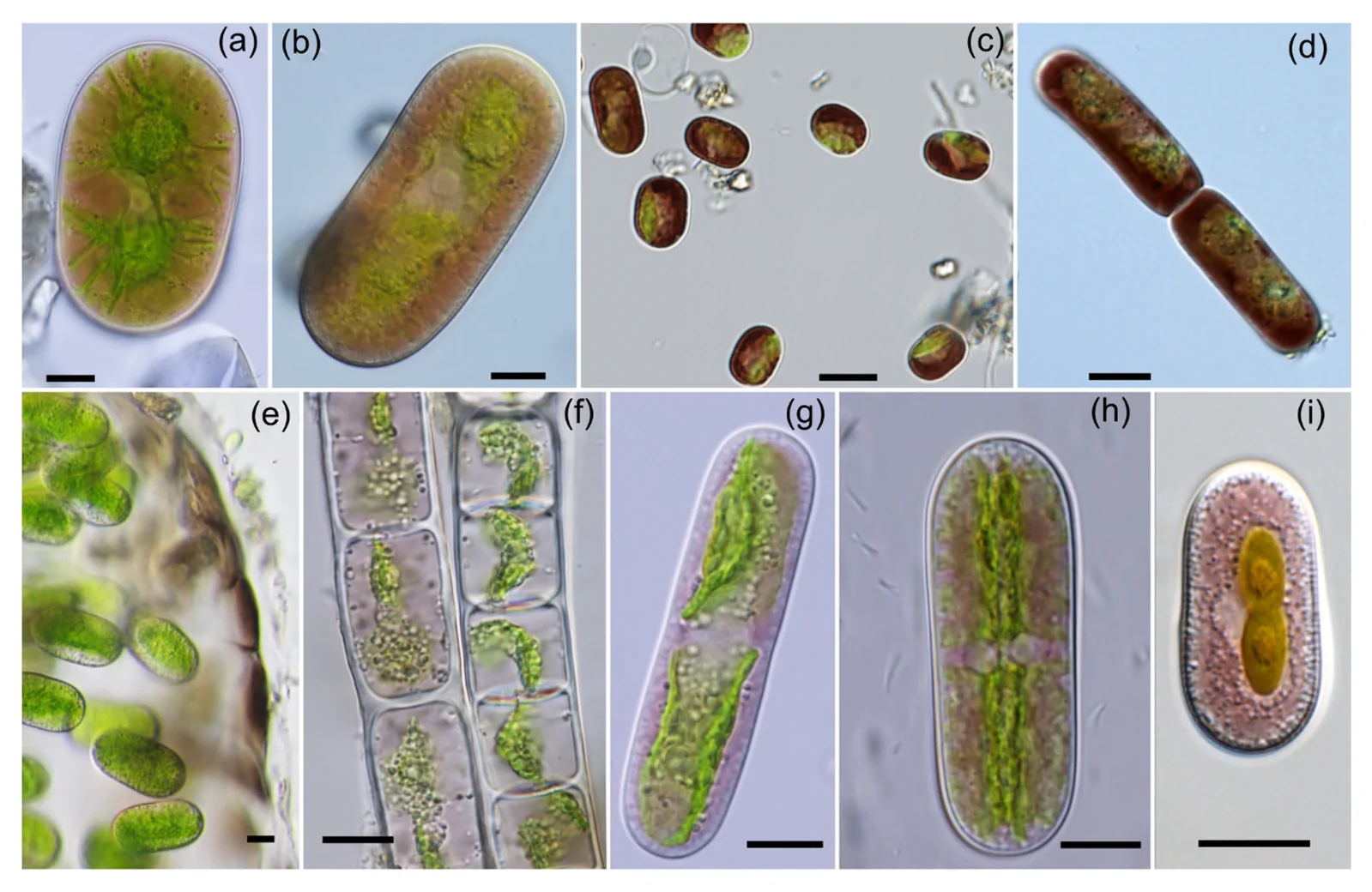
Light micrographs of CGA field cells collected for demonstrating the pigmented vacuoles or secreted pigments. (**a**) *Cylindrocystis* sp. from a glacier ice surface, Colombia, (**b**) *Cylindrocystis* sp. from a glacier ice surface, Svalbard, (**c**) *Ancylonema alaskanum* from a glacier ice surface, Austria, (**d**) *Ancylonema nordenskioeldii* from glacier ice surface, Svalbard, (**e**) *Serritaenia* sp. from mucilage on *Betula* tree bark, Northern Czech Republic. Note that the dark pigment was secreted into the mucilage to the right, (**f**) *Zygogonium ericetorum* from a spring puddle in the Austrian Alps, (**g**) *Ancylonema palustre*. from a very shallow puddle on peatbog, Southern Czech Republic, (**h**) *Cylindrocystis* sp. from a minerotrophic fen, Northern Czech Republic, (**i**) *Mesotaenium chlamydosporum* var. *violascens* from mucilage on a wet rock, Eastern France. Scale bar: 10 μm

For decades, it has been well known that in many genera of CGAs, such as *Cylindrocystis, Mesotaenium, Zygogonium* or *Actinotaenium*, cells can produce secondary pigments that cause striking purple or brownish vacuolar pigmentation (Fig. 2). This phenomenon is quite uncommon in other freshwater microalgae. Likewise, the zygospore cell walls of various CGAs can exhibit remarkable blue or brown colours, and aromatics inside the mesospore layer may be responsible (Permann et al. 2021a). A third aspect is the presence of uncoloured but highly abundant gallotannins in the genus *Spirogyra* (Han et al. 2012). The phenolic nature of these specialized compounds has long been discussed, and analytical breakthroughs of both Newsom & van Breemen (2012) and Remias et al. (2012b) enabled structural elucidations to reveal their chemical constitutions. The former study identified the purple phenol of *Zygogonium ericetorum*: A highly branched glucose polymer in combination with traces of ferric iron complexes and covalently bound gallic acid groups was described, with the actual colour caused by the ferric gallate coordination-complexes. In the second work, purpurogallin carboxylic acid-6-*O*-β-d-glucopyranoside was found as the main phenol in *Ancylonema alaskanum*. Blooms of both species cause macroscopic, exposed discolorations in either melting ice surfaces (Fiołka et al. 2025) or acidic, damp semiterrestrial habitats (Stancheva et al. 2025).

The ecophysiological role of CGA phenolics is primarily considered to be either protection against UV and excessive VIS irradiation of the nucleus and chloroplasts (to reduce DNA mutation rates and present photoinhibition of photosystems), or as a defensive compound against herbivores. Other potential roles such as desiccation tolerance, radical scavenging or chemical defence are also under discussion. Similar but non-pigmented phenols found in *Spirogyra* or *Mougeotia* can be considered bioactive compounds (Phinyo et al. 2024; Sitthiwong et al. 2024). With their complex structure, some CGA phenolics are highly distinctive compounds from a chemotaxonomic point of view and probably unique within the plant kingdom.

Several studies have adopted an ecophysiological approach to try to elucidate the triggers for phenol accumulation. For instance, Pichrtová et al. (2013) observed an elevated level of soluble phenols in *Zygnema* strains following seven days of UV exposure. Remarkably, certain species accumulate phenols in vacuoles and can actively secrete them into the extracellular space on demand. The most sophisticated strategy yet described was exhibited by an aeroterrestrial species of *Serritaenia*, which secreted a brownish pigment in the direction of an incoming light and UV source (Busch and Hess 2022a; Busch et al. 2024b). The role of these CGA phenols in providing protection against irradiation is evident, but this hypothesis has not yet been thoroughly tested. With regard to temperature dependence, soluble phenol accumulation in mesophilic *Zygnema* species showed no correlation within a temperature range of 15–25 °C (Permann et al. 2022b). In contrast, Han et al. (2012) observed a significant increase in gallotannins in *Spirogyra varians* during a temperature change from 20 to 4 °C.

Using a combination of Raman spectroscopy (RS) and colorimetric assays, Procházková et al. (2025) found strong evidence that the dark vacuoles of the glacier ice alga *Ancylonema alaskanum* do not only contain purpurogallin-derivatives (Remias et al. 2012b). Instead, and unexpectedly, significant amounts of iron are also present. Consequently, dark brown Fe-complexes of this phenol contribute significantly to the strong spectral UV- and VIS-absorption of this glacier ice algae. The isolated pure purpurogallin is only yellowish (Procházková et al. 2025). This is of significant ecological consequence far beyond the alga’s intracellular strategy of coping with excessive irradiation, since dark blooms of glacier ice algae lead to surface albedo reductions over vast regions of the western Greenland Ice Cap, accelerate melting rates and thus speeding up the global sea-level rise (Williamson et al. 2020).

Interestingly, despite the fact that many marine and fresh-water micro- and macroalgae, including the streptophytic lineage, accumulate Mycosporine-like Amino Acids (MAAs), they have never been reported from any CGA (Holzinger and Pichrtová 2016), even though they act as powerful UV-B protectants. Nasciemento & Tattini (2022) argue that during the process of terrestrialisation, the nitrogen-containing MAAs were substituted with biosynthetically “cheaper” phenolics, driven by lower nitrogen availability in the new terrestrial habitats. Moreover, despite being nitrogen-free compounds, secondary carotenoids like the red astaxanthin do not occur in CGAs either. Finally, the flavonoids, well-known from land plants, have similar cellular functions, but only a few reports of their occurrence in CGAs (*Penium margaritaceum*, Jiao et al. 2020). Unfortunately, that study neither found all the necessary genes for synthesis in the genome, nor were any indications given about actual intracellular abundances. Nevertheless, the absence of complex flavonoids or nitrogen-expensive MAAs does not imply a lack of chemical defence. Rather, it highlights a metabolic shift toward a specialized phenylpropanoid ‘scaffold.’

In summary, from what is currently known, it seems that CGAs developed their own kind of characteristic phenols based on glucogallin derivatives. Table 1 shows a qualitative overview of phenol classes so far found across orders and genera, based on literature and preliminary observations of the authors. Note, the abundant presence of phenols is not necessarily linked to vacuole pigmentation, i.e. in the case of tannins in the Spirogyrales.

## Conclusions

CGA have been well known since the onset of light microscopy keeping the beautiful large desmid cells in mind. In an evolutionary point of view, they were soon recognized as a distinct, natural group within the green algae due to the striking sexual process of meiotic fusion without flagellate stages but a characteristic conjugation channel instead. With the onset of the molecular age, the morphological complex stoneworts were replaced by the Zygnematophyceae as the closest existing relatives to land plants. The morphological simplicity of the latter is regarded as a reduction phenomenon for surviving adverse climatical ages on Earth, but their more complex genomes including horizontal gene transfer events, in contrast to non-streptophytic green algae, fosters their ecological position in the onset of conquering the land.

Future research on CGA will depend on expanding laboratory cultures in which zygospore formation can be easily induced. This will facilitate the identification of new synapomorphic criteria during meiosis and the respective reduction cleavages. The asynchrony between chloroplast and cell cleavage still lacks an evolutionary explanation. The physiological differences between the five new orders await elucidation. As more genomes and transcriptomic studies will become available in the coming years, our understanding of how microalgae cope with environmental stresses and how they maintain their dominance in extreme niches will increase. Specialised metabolites such as complex phenolic compounds require further chemical characterisation and ecophysiological testing. Understanding the regulation and secretion of specialized pigments remains a key frontier in algal ecophysiology, offering insights into both ancestral stress-response mechanisms and current global environmental changes. Lastly, the taxonomy of polyphyletic genera particularly within the Mesotaeniaceae family - which houses *Mesotaenium endlicherianum*, the species closest to land plants - remains a priority for a consistent streptophyte phylogeny.

## Figures and Tables

**Table 1:** A qualitative scheme showing the occurrence of phenol classes in different CGA orders and genera and the predominant vacuole colour (if present). Glucogallin seems to be the basic compound, present in all so far investigated genera, it has one spectral absorption maximum in the UV. Glycolyzed “Tannins” seem to be exclusive to the Spirogyrales. The “Complex Phenols” are larger compounds (polyphenolic) and defined by more than one UV spectral absorption maximum. “Metal-Complexes” of phenols have two or three short-wave absorption maxima and a significant, additional long-wave absorption in the VIS-range, causing striking pigmentation. True flavonoids have been found so far only in the desmid *Penium*. For the Spirogloeales, no analytical data are available yet. The scheme is based on the literature and own preliminary analytical results. n.d., not detected

Genus	Penium	Spirogyra	Zygnema	Cylindrocystis	Ancylonema	Zygogonium	Serritaenia
Order	Desmidiales	Spirogyrales	Zygnematales	Zygnematales	Zygnematales	Zygnematales	Serritaeniales
Color	no	no	no (yellow)	(brownish)	brownish	purple	(brownish)
Glucogallin	likely	yes	yes	yes	yes	yes	yes
Tannins	?	yes	no	no	no	no	no
Complex phenols	?	n.d.	yes	likely	yes	yes	likely
Metal-complexes	?	n.d.	likely	likely	yes	yes	likely
Flavonoids	yes	n.d.	n.d.	n.d.	n.d.	n.d.	n.d.
Reference	Jiao et al. (2020)	Nishizawa et al. (1985); own data	Pichrtová et al. (2013); Permann et al. (2022b)	own data	Procházková et al. (2021, 2025); Remias et al. (2012b)	Newsome & van Bremen (2012); own data	Busch and Hess (2022b); own data

## Data Availability

No datasets were generated or analysed during the current study.
